# Supplementary material for: Polystyrene as Graphene Film and 3D Graphene Sponge Precursor
Source: Nanomaterials (Basel). 2019 Jan 16;9(1):101. doi: 10.3390/nano9010101 (PMC6358832; doi:10.3390/nano9010101)
Supplement: Supplementary file 1 [file nanomaterials-09-00101-s001.pdf]

**Supporting information**

# Polystyrene as Graphene Film and 3D Graphene Sponge Precursor

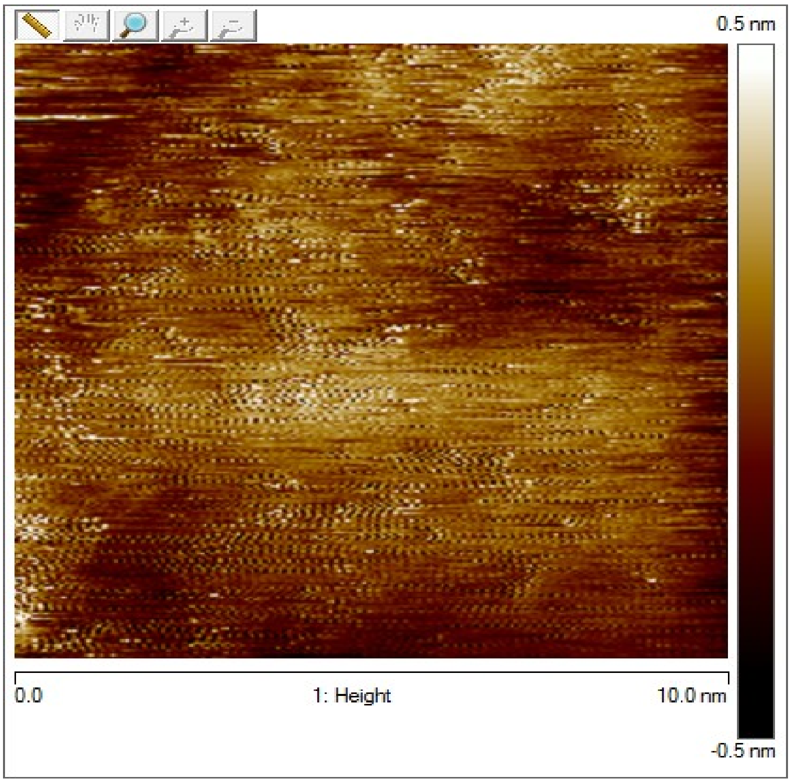


Figure S1. High resolution scan tunneling microscopy (STM) of defective graphene using polystyrene as precursor
